# Superior Pre-Osteoblast Cell Response of Etched Ultrafine-Grained Titanium with a Controlled Crystallographic Orientation

**DOI:** 10.1038/srep44213

**Published:** 2017-03-07

**Authors:** Seung Mi Baek, Myeong Hwan Shin, Jongun Moon, Ho Sang Jung, See Am Lee, WoonBong Hwang, Jong Taek Yeom, Sei Kwang Hahn, Hyoung Seop Kim

**Affiliations:** 1 Department of Materials Science and Engineering, Pohang University of Science and Technology (POSTECH), Pohang, 37673, Republic of Korea; 2 Korea Institute of Materials Science, Changwon, 51508, Republic of Korea; 3 Department of Mechanical Engineering, Pohang University of Science and Technology (POSTECH), Pohang, 37673, Republic of Korea; 4 Center for High Entropy Alloys, Pohang University of Science and Technology (POSTECH), Pohang, 37673, Republic of Korea

## Abstract

Ultrafine-grained (UFG) Ti for improved mechanical performance as well as its surface modification enhancing biofunctions has attracted much attention in medical industries. Most of the studies on the surface etching of metallic biomaterials have focused on surface topography and wettability but not crystallographic orientation, i.e., texture, which influences the chemical as well as the physical properties. In this paper, the influences of texture and grain size on roughness, wettability, and pre-osteoblast cell response were investigated *in vitro* after HF etching treatment. The surface characteristics and cell behaviors of ultrafine, fine, and coarse-grained Ti were examined after the HF etching. The surface roughness during the etching treatment was significantly increased as the orientation angle from the basal pole was increased. The cell adhesion tendency of the rough surface was promoted. The UFG Ti substrate exhibited a higher texture energy state, rougher surface, enhanced hydrophilic wettability, and better cell adhesion and proliferation behaviors after etching than those of the coarse- and fine-grained Ti substrates. These results provide a new route for enhancing both mechanical and biological performances using etching after grain refinement of Ti.

Metallic biomaterials have been advanced to replace the hard tissue of a deteriorated body as the aging of populations is rapidly accelerating worldwide. For several decades, there has been increasing interest in the studies of metallic biomaterials, mainly stainless steels, Co-based alloys, and Ti alloys, for artificial joints, dental implants, and so on^1^.

However, it is known that many alloying elements of commercial materials, such as Ni, Co, V, and Al, have a cytotoxicity which can induce necrosis, allergic responses, carcinogenic effects^2^, and neuronal damage^3^. Thus, pure Ti has gained much attention in academia and medical industries due to its non-toxicity and superior mechanical properties^2^. Pure Ti has good fatigue resistance and a relatively low elastic modulus which can reduce the shielding stress in an implant^4^,^5^. In particular, a chemically inert oxide layer of the Ti surface contributes to its superior corrosion resistance and exceptional osseointegratibility for hard tissue replacement materials^6^,^7^.

However, the mechanical properties, in particular, the long-term loading strength, of pure Ti are not as good as the above mentioned commercial alloys. For this reason, many efforts using grain refinement by severe plastic deformation (SPD) have been done to overcome the limitation of pure Ti. The SPD process, a “top-down” nanocrystallization approach, creates nanostructured or ultrafine-grained (UFG) materials from coarse-grained materials^8^,^9^,^10^. Several studies have shown a higher tensile strength for UFG Ti processed by high-pressure torsion (HPT) compared to conventional Ti-6Al-4V^11^ and sufficiently high tensile and fatigue strengths of UFG Ti processed by equal-channel angular pressing (ECAP)^12^,^13^. Consequently, UFG Ti has potential applicability for medical materials due to its sufficient strength and fatigue properties as well as its excellent biological properties.

Recent studies have reported that UFG Ti enhances the response between cells and the substrate surface. In the case of pre-osteoblast^14^,^15^ and fibroblast^16^,^17^ cells, adhesion and proliferation of the cells with higher fibronectin expression have been enhanced in the UFG Ti substrate compared with commercial Ti-6Al-4V. Improvement of fibronectin attachment, which is related to osteoblast adhesion^18^, on the UFG Ti substrate has been reported for *in vivo* as well as *in vitro* conditions^19^. UFG Ti also promotes osteoblast differentiation and increases bone integration effectively^20^ due to the effects of the formation of nano-defects, surface energy, wettability, and the oxide layer of the UFG pure Ti.

Surface treatments, e.g., cleaning, etching, or coating, of medical parts are preferably performed in medical industries to improve the insufficient bioactivity of metallic biomaterials. Surface cleaning is generally required to remove the native surfaces of metallic biomaterials which contain a non-uniform oxide layer, unnecessary contaminants, structural defects, and undesired reaction films^21^. Wet chemical etching is one of the effective ways to remove the native surface layer and to generate a rough surface with a uniform oxide layer. Etching methods, non-coating processes, have less risk of debris and particles and improve the biocompatibility through the evolution of the substrate surface for roughness and wettability^22^. In addition, etching has potential for a combination of hydroxyapatite (HA) coating, titanium plasma spraying (TPS), and other surface treatment methods^23^.

Most of the research on the cell response of UFG Ti for biomaterials have focused on roughness, wettability, and the oxide layer of the surface rather than on the surface energy, in particular, related to the crystallographic orientation, i.e., the texture^24^. The crystallographic orientation reported recently in a few studies is related to only the HA coating and machined surface despite the fact that the crystallographic orientation significantly influences the physical and chemical properties of a material^25^,^26^. In particular, the effect of the crystallographic orientation on the etching behavior for biomaterials has not been investigated as far as the authors know although it is already well known that texture controls most of the material properties such as the mechanical, electrical, and magnetic properties^27^.

In this paper, we investigated the effects of the crystallographic orientation of Ti on the roughness and wettability of the surface during the etching treatment after grain refinement through HPT. Its effect on pre-osteoblast cell responses *in vitro* after etching was evaluated. In addition, we examined the relationship between orientation, roughness, wettability, and cell behavior for coarse-, fine-, and ultrafine-grained pure Ti after hydrofluoric etching treatment.

## Results

### Microstructure and mechanical properties

Table 1 shows the grain sizes and mechanical properties of the Ti samples. The mean values ± standard deviation are presented. The HPT process reduced the grain size from 118 ± 49 μm of the as-received Ti to approximately 100 nm. After annealing at 550 °C for 2 h, the grain size increased from 100 nm of the HPT-processed Ti to 3 ± 1 μm (Supplementary Fig. S1). The HPT-processed Ti has an improved ultimate tensile strength (UTS) value and sufficient total elongation compared with the Ti-6Al-4V^5^,^28^.

**Figure 1:**
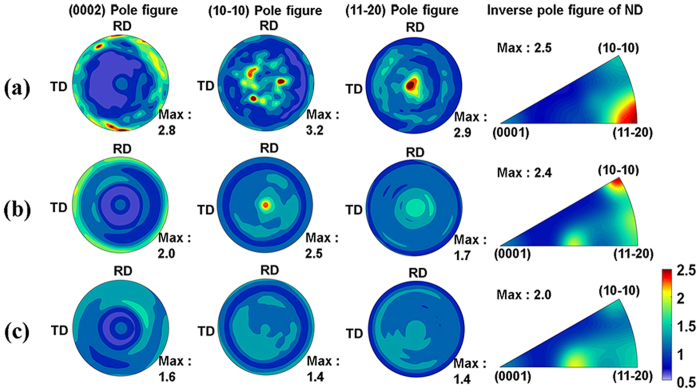
(0002), (10-10), and (11-20) pole figures and inverse pole figures of ND of CP-Ti samples. (**a**) as-received; (**b**) after HPT 5 GPa and 5 turns; and (**c**) after HPT + annealed at 550 °C for 2 h.

The examined samples had different crystallographic orientations as shown in Fig. 1. The inverse pole figures of the normal direction (ND) suggest that the evolution of the orientation occurred during the HPT process and the following annealing-processes. The peak intensities of the as-received, the HPT-processed, and the annealed HPT-processed substrates exist in the (11-20), (10-10), and (11-23) poles, respectively. The annealed substrate had a weak crystallographic orientation (Fig. 1c). The volume fractions of the basal fiber for the as-received, the HPT-processed, and the annealed HPT-processed substrates are 2.83%, 2.76%, and 2.89%, respectively. There is no significant difference in the volume fractions of the basal fiber.

### Surface characterization

**Figure 2:**
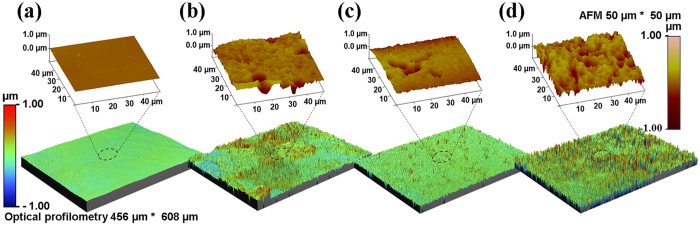
Surface topography measured using AFM and optical profilometry. (**a**) as-received; (**b**) eCG; (**c**) eFG; and (**d**) eUFG.

The as-received coarse-grained (eCG), the HPT-processed and annealed fine-grained (eFG), and HPT-processed UFG (eUFG) substrates were HF etched for 20 min and examined with atomic force microscopy (AFM) and optical profilometry for surface topography and roughness measurements shown in Fig. 2. The 3D profiles are presented from a small area (50 × 50 μm^2^) to a large area (456 × 608 μm^2^), and the roughness parameters, the mean value ± SD, with the values for the surface area difference are presented in Table 2. After etching, the roughness values differed between the groups. Particularly, the eUFG had the highest roughness value and largest surface area. The average and root mean square roughness value of the eCG was higher than that of the eFG (*p* < 0.001).

**Figure 3:**
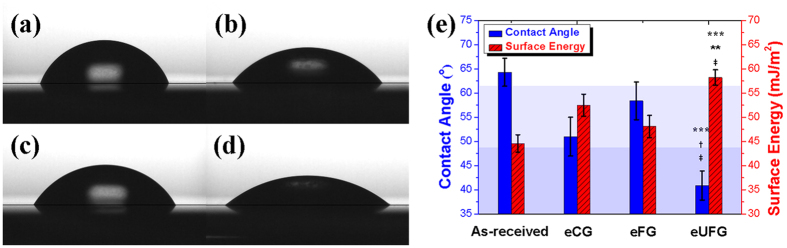
Snapshots of the drop shape on surfaces and contact angle values and surface energy. (**a**) as-received; (**b**) eCG; (**c**) eFG; and (**d**) eUFG. (**e**) represents the mean value of contact angle with calculated surface energy of each sample. ^***^p < 0.001 compared to the as-received substrates; ^**^ if *p* < 0.01, and ^†^ if *p* < 0.001 compare with the eCG substrates; and ^‡^*p* < 0.001 with respect to the eFG substrates.

Figure 3 shows the drop shape images, contact angle values, and surface energy on different surfaces. The values for the contact angle, 48–62° and 35–50°, indicate a moderately wettable and higher hydrophilic surface, respectively. The acid etching treatment on the Ti generated relatively hydrophilic surfaces: the surface of the eUFG had a meaningfully lower contact angle than that of the other surfaces (*p* < 0.001). Thus, the calculated surface energy of the eUFG was higher than those of the as-received, eFG (*p* < 0.001), and eCG (*p* < 0.01) surfaces. The surface of the eCG had a lower contact angle and higher calculated surface energy than that of the eFG surface (*p* < 0.05).

### Cell adhesion and proliferation rate

**Figure 4:**
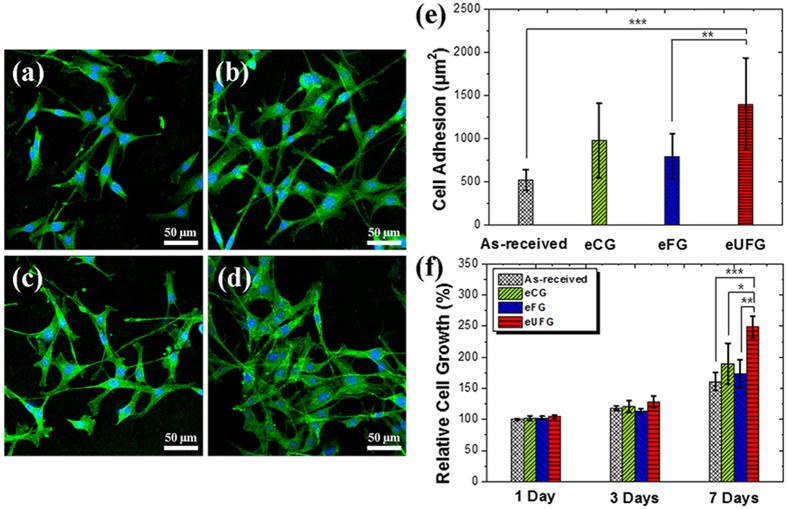
Pre-osteoblast cells spreading on the surfaces and proliferation rate. Fluoresence images of F-actin (green) and nucleus (blue) stained cells on (**a**) the as-received, (**b**) eCG, (**c**) eFG, and (**d**) eUFG substrates after 3 days. (**e**) The average cell adhesion area on each substrate by measuring the region of F-actin fluorescence using image J software (^***^p < 0.001 and ^**^p < 0.01). The fluorescence images of F-actin were used false color from red to green, and merged with the nucleus fluorescence images. (**f**) Proliferation rate of the pre-osteoblast cells with a reference of the as-received CP-Ti substrate (^***^p < 0.001, ^**^p < 0.01, and ^*^p < 0.05).

Figure 4 shows images of pre-osteoblast cells spreading on the surfaces from each group. Cells on the eUFG surface had a well-extended morphology and developed a strong linkage of more clear and spread fibers compared with the as-received, eCG, and eFG surfaces after 3 days (Fig. 4a–d). The eUFG substrate had a higher value for the average area per one cell than that of the as-received (*p* < 0.001), eCG (*p* > 0.05), and eFG (*p* < 0.01) substrates (Fig. 4e).

As shown in Fig. 4f, the proliferation rate of the cells was monitored for 7 days of culturing. After 7 days, cells on the eUFG surface had a higher cell proliferation rate than those on the as-received (*p* < 0.001), eCG (*p* < 0.05) and eFG (*p* < 0.01) surfaces. There was no significant difference in the attachment of cells between the as-received, eCG and eFG (*p* > 0.05) although the eCG had a slightly higher mean value after 7 days.

**Figure 5:**
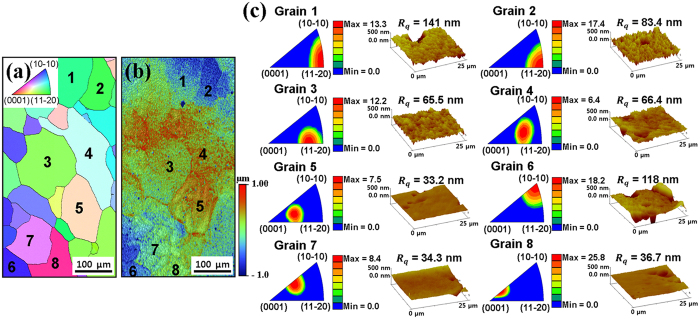
The orientation and surface roughness of each grain after etching. (**a**) EBSD micrographs and (**b**) optical profilometry images in the same region on the as-received substrate. Each number points out each grain. (**c**) Inverse pole figures of normal direction and surface topography (scan area 30 × 30 μm^2^) with R_q_ values of each grain.

The orientation and surface roughness of each grain after etching are shown in Fig. 5. The regions from the electron back-scatter diffraction (EBSD) micrographs and optical profilometry images are matched (Fig. 5a,b). The peak intensities of the inverse pole figures for the ND and the root mean square roughness values of the eight major grains were examined shown in Fig. 5c.

**Figure 6:**
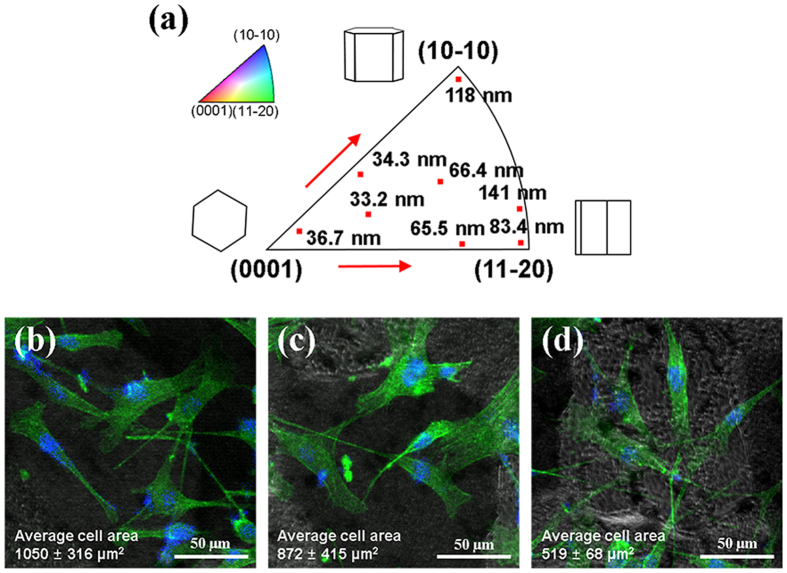
Relationship between the orientation and surface roughness after etching and cell spreading on the surfaces with different roughness. (**a**) Inverse pole figure of normal direction with roughness measured at specific orientations (red dot). Confocal microscopic images of the F-actin (green) and nucleus (blue) stained cells on (**b**) grain 1, (**c**) grain 3, and (**d**) grain 5 after 3 days. Grain numbers are indicated in Fig. 5. The fluorescence images of F-actin were used false color from red to green, and merged with the nucleus fluorescence images.

Figure 6a shows the interrelation between the orientation and roughness value during etching. The roughness increased as the peak intensity moves further away from the basal pole (0001). Cells on the rough surface spread well and developed their fibers widely as a dendritic shape (Fig. 6b,c); however, cells on the smooth surface formed an aligned shape rather than the dendritic shape (Fig. 6d). Grain 1 had a higher value for the average area per one cell than that of grain 3 (*p* > 0.05) and grain 5 (*p* < 0.05). There was a tendency for the cell adhesion area to increase with the surface roughness.

## Discussion

HPT is a representative SPD process inducing significant grain refinement in bulk materials by extremely large shear strains with hydrostatic pressure^11^. Pure Ti, which is a hexagonal-close-packed (HCP) metal, has complex deformation modes. HCP materials have several different dislocation slip systems, such as basal {0001} <1-210>, prismatic {10-10} <11-20>, and pyramidal {10-11} <-12-10> and deformation twinning systems^29^. The operating slip and twin systems depend on deformation conditions, such as the stress state, strain rate, and temperature^30^. During the HPT of pure Ti at room temperature, prismatic and pyramidal slip systems occur preferentially^31^, and grain refinement effectively occurs. The UFG structure leads to superior strength and sufficient ductility of the Ti due to the increased fraction of high-angle grain boundaries. High-angle grain boundaries enhance strength because they block dislocation glide, and a small grain size promotes grain boundary sliding and grain rotation which increase the ductility and superplasticity^32^. For these reasons, the HPT-processed Ti has a UFG structure and higher UTS and total elongation values compared with the commercial material, Ti-6Al-4V.

Plastic deformation processes generate a variety of crystallographic orientations depending on the initial texture, deformation mode, and history. For example, ECAP developed a prismatic plane in the HCP structure with an initially non-random texture^33^ while theoretically, a nearly prismatic plane was developed during simple shear on the HCP structured materials^29^. In this study, texture evolution in the HPT-processed Ti was carefully investigated: the second order prismatic plane (11-20) in the as-received state evolved to the prismatic plane during the HPT process. This indicates that the activity of the prismatic slip mode is the primary slip system during the HPT process because the HPT deformation mode is dominantly shear. It was reported that annealing can reduce the dislocation density and residual stress with the development of a basal texture, i.e., annealing texture, in UFG Ti^34^. There was no significantly developed basal plane in the annealed substrate; however, the dominant intensity of the ND was close to the basal pole with a weak texture. This indicates that the annealing treatment removed the fiber texture in the UFG Ti.

The etched surface of the HPT-processed UFG Ti substrates had a higher roughness value and better wettability and pre-osteoblast cell behavior than that of the coarse- and fine-grained substrates. It has been reported that the wettability of a rougher surface substrate is enhanced when compared with a smooth surface under a hydrophilic surface condition^35^. In the present study, when the surface was the rougher, the value for the contact angle was lower. That is, when the roughness value increases, the wettability of the surface is enhanced because the surface area increases with the surface roughness. Hydrophilic surfaces have an important role in cell activity because the adsorption of the extracellular matrix (ECM) proteins, which promote cell attachment and proliferation, is enhanced on surfaces that are more hydrophilic^36^,^37^. It is well known that ECM proteins affect cell signaling, nuclear organization, and cytoskeletal formation by binding integrin^38^. For these reasons, the biological response to a substrate is associated with its surface wettability which is related to the oxide layer, roughness, and surface energy. Recent studies have suggested that a hydrophilic (contact angle = 35–50°) surface has maximum endothelial cell adhesion due to the increased adsorption of proteins and that a surface with moderate wettability (contact angle = 48–62°) also has enhanced biocompatibility^39^,^40^,^41^. Additionally, it is known that the higher wettability of the UFG substrates contributes by enhancing the adsorption of proteins, cell adhesion, proliferation and differentiation^20^,^37^. Our results show that the HPT-processed and etched Ti substrate, which has a hydrophilic surface (contact angle = 40.9 ± 3.0°), has better adhesion and proliferation of pre-osteoblast cells than those of the unetched and etched coarse- and fine-grained Ti substrates which have a moderately wettable surface. Therefore, it is clear that a hydrophilic surface is advantageous in terms of cell adhesion and proliferation when compared with a moderately wettable surface.

It has been reported that a thick, uniform, and inert oxide layer and pore structure, which promote wettability and biocompatibility, were effectively developed after etching Ti substrates in a nitric and hydrofluoric acid mixed solution^42^,^43^. Additionally, it has been reported that the thickness and composition of the oxide layer practically are not different between coarse-grained and nanostructured Ti with the same etching condition although a difference in the roughness does exist between the coarse-grained and nanostructured Ti^21^,^44^. It was reported that the grain size is a primary factor affecting roughness during etching^22^. In another study, the effect of the grain orientation on the electrochemical behavior was more significant than that of the grain size^45^. On the other hand, the orientation effect on surface roughness during etching treatment has not been investigated yet. In the present study, interestingly, the coarse-grained substrate has a higher roughness and better wettability than that of the fine-grained substrate after the etching treatment. It should be noted that plastic deformation and heat treatment generate a deformation texture and recrystallization texture, respectively^27^, as mentioned before. That it, not only the grain boundaries but also the crystallographic orientation of the surface contributes to the surface energy affecting the etching behavior.

The highest planar atomic density of the basal plane results in the lowest surface energy in a hexagonal close-packed crystal. It was reported that surface energies of the basal plane (0002), first order prismatic plane (10-10), and second order prismatic plane (11-20) in Ti are 988, 1049, and 1132 ergs cm^−2^, respectively^46^. Recent studies have confirmed that the basal planes of crystals in polished Ti substrates promote cell attachment^47^ and (11-20) single-crystal Ti substrate of polished surface showed higher pre-osteoblast cell attachment than the other planes^48^; on the other hand, we expect that the basal plane of the lowest surface energy is less vulnerable to etching than that of the other planes. That is, the basal planes develop smooth surface during etching; hence, the cell responses on a rough substrate of non-basal planes are improved because the wettability of a substrate with a rougher surface is enhanced compared with a smooth surface under a hydrophilic surface condition^37^. To test our idea, we examined the etching effect of the coarse-grained Ti by evaluating the orientation, roughness, and cell response, including the adhesion area and cellular shape, for each grain. Our results are in accordance with the results of Bahl *et al*.^25^ that the lower deviation of the basal plane from the ND towards the transverse direction leads to enhanced corrosion resistance. The inverse pole figure of the ND with the roughness measured at a specific orientation shows a meaningfully etching effect on the (10-10) and (11-20) orientations. According to the roughness results, the fibers of the pre-osteoblast cells spread better on rougher surfaces. For this reason, the coarse-grained substrate, which had an orientation relatively far from the basal pole, had a rougher surface, enhanced wettability, and better cell response than that of the fine-grained substrate despite their large grain size and insignificant grain boundaries volume in this study. These results show that the roughness, wettability, and cell response are strongly influenced by the orientation of the substrates as well as the grain size after the etching treatment.

A previous study on Ti reported that high residual stresses are characteristic of UFG materials processed by SPD^49^ and deteriorate corrosion resistance^50^. Therefore, we suggest that the UFG of the HPT-processed Ti substrate, which has a higher surface energy with many grain boundaries and an orientation far from the basal pole, is effective in etching for biomedical applications.

In this study, the HPT-processed and HF-etched UFG Ti substrates had a higher roughness, enhanced wettability, and better cell adhesion and proliferation behavior. These results suggest that the surface of this substrate has a higher surface energy than the as-received or the HPT-processed Ti with annealed substrates. The prismatic peak intensity of the ND and the numerous grain boundaries contribute to the higher surface energy of the HPT-processed Ti. The UFG Ti well develops a higher roughness that contributes to a better surface wettability during the HF-etching treatment, and subsequently, the better wettability of the surface leads to an enhanced cell response compared with the as-received and HPT-processed Ti with annealed surfaces. Furthermore, the superior mechanical properties of the HPT-processed Ti is expected to result in the downsizing of implants which will subsequently attenuate or possibly eliminate biological problems. Further studies are needed to determine the orientation effect on the combined treatment of etching and other process, such as HA coating and TPS, for UFG Ti used in biomedical applications. Studies on new deformation and heat treatment processing routes to develop more (11-20) textures are underway. This research direction will provide new opportunities to develop better metallic biomaterials.

## Conclusions

In this study, we investigated the crystallographic orientation of Ti substrates in terms of the roughness, wettability, and cell response *in vitro* after the etching treatment. The roughness of the surface was significantly increased for the orientation far from the basal pole during the etching treatment and promoted the cell adhesion tendency of the rougher surface. The HPT-processed UFG Ti substrate, which had a higher surface energy based on the effect of the orientation and numerous grain boundaries, had a rougher surface, enhanced hydrophilic wettability, and better cell adhesion and proliferation behavior after etching compared with the coarse- and fine-grained Ti substrates. Therefore, the UFG pure Ti substrate fabricated by HPT followed by the etching process might have the potential for long-term loading biomedical applications because it provides superior surface properties as well as exceptionally superior mechanical properties. Additional studies on the crystallographic orientation effect on combined treatments are needed for wide commercial applications.

## Materials and Methods

### Materials and grain refinement

ASTM B348 commercial purity grade 2 Ti (ATI Inc., USA) was used as-received (annealed at 704 °C for 2 h). Disk-shaped specimens with a diameter of 10 mm and a thickness of 1.5 mm were prepared by wire cutting for HPT operations. The HPT process was performed at 5 GPa and 5 revolutions at room temperature which proves the saturated grain refinement of the Ti. To obtain fine grain sized samples, i.e., between UFG and coarse ones, an additional annealing process (550 °C for 2 h) was applied to the HPT-processed UFG specimens.

For the analysis of mechanical properties, tensile specimens (gauge length 1.5 mm, gauge width 1.0 mm, and gauge thickness 0.7 (the HPT-processed and annealed samples) and 1.0 (the as-received) mm) were obtained from 2.5 mm away from the center of the disk-shaped specimens. Tensile tests were carried out at a strain rate of 10^−3^ s^−1^ at room temperature with a universal testing machine (Instron 1361, Instron Corp., USA). Strain distributions of the samples during the tensile test were examined using digital image correlation with a vision strain gage system (ARAMIS v6.1, GOM Optical Measuring Techniques, Germany).

The measurement of crystallographic orientation was performed with Co Kα radiation in an X-ray diffractometer (D8-Discover, BRUKER AXS, Germany) for texture analysis. A set of five pole figures, (10-10), (0002), (10-11), (10-12), and (10-13), was measured on the normal plane. The orientation distribution function (ODF) was calculated with the MTEX toolbox. Pole figures, inverse pole figures, and the volume fraction of the basal fiber with a 15° orientation spread in Euler’s angles were derived using the ODFs for texture analysis. The specimens were polished down to P-4000 using sandpaper with ethanol.

### Surface treatment

Wet chemical etching can remove a nonuniform native surface layer and create a uniform oxide layer and rough surface. In particular, a mixed nitric and hydrofluoric acid solution can effectively form pore structures on the Ti surface. Using the mixed solution, roughness can be easily controlled by the etching time and concentration. The ratio of nitric acid to hydrofluoric acid should be maintained at 10:1 to prevent hydride formation because unwanted hydride formation leads to metal embrittlement on the surface^21^.

For the cell culture experiments, all of the disks were prepared with an 8 mm diameter and 0.7 mm thickness. The specimens were polished down to 0.25 μm using sandpaper with ethanol and a 0.05 μm colloidal silica solution to achieve a mirror surface which was subsequently etched in a mixture of acids consisting of 90 volume% HNO_3_ (60 mass%) and 10 volume% HF (48 mass%) for 20 min at room temperature. After the etching, the specimens were washed with deionized water to reach a neutral PH for 10 s and then washed with 99.9 vol% ethanol in an ultrasonic bath.

Surface topography and roughness parameters were measured with AFM and optical profilometry. AFM (Veeco Dimension 3100 and Nanoscope V7.0, Veeco, USA) was used to measure surface topography of each specimen at the nano and micro-level with a scan size 50 × 50 μm^2^. The microscope mode was tapping with an aspect ratio of 1.0 at a scan rate of 0.337 Hz. Optical profilometry (Wyko NT1100, Veeco, USA) was used to scan 456 × 608 μm^2^ areas and to analyze the roughness parameters which were calculated automatically by the manufacturer’s software. Four specimens from each group were examined to evaluate the average roughness parameters using optical profilometry by considering a large measurement area. Three specimens from each group were measured to calculate the difference in the surface area using AFM.

Wettability was evaluated by measuring the contact angle (Smartdrop, Femtofab, Republic of Korea) which takes into consideration the volume of the solution. Deionized water, 5 μl, was dropped onto each specimen with an auto-pipette (Finnpipette Novus, Thermo Scientific, USA) at 25 °C. The average contact angles were measured by the sessile drop method from four specimens of each group. The solid surface energies were calculated using the following Young’s equation^51^:


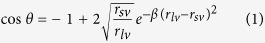


where 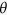, 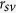, and *β* are the advancing contact angle (radian), the solid surface free energy, and the constant value (0.0001247 m^2^/mJ), respectively. 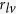 is the liquid surface tension between pure water and air (72.0 mJ m^−2^ at 25 °C)^52^.

### Bio characterization

Osteoblast precursor cells, MC3T3-E1, provided by the Korean Cell Line Bank (KCLB, Republic of Korea) were cultured in alpha minimum essential medium (α-MEM, WelGENE Inc., Republic of Korea) with 10% fetal bovine serum (Gibco, USA), 100 U ml^−1^ penicillin (WelGENE Inc., Republic of Korea) and 100 μg ml^−1^ streptomycin (WelGENE Inc., Republic of Korea) at 37 °C in a humidified atmosphere of 5% CO_2_.

Before the cell culture, all the samples were sterilized by immersing in 70% ethanol for 10 min. After drying under UV lamp in the clean bench with air flow for 2 h. MC3T3-E1 cells at a density of 1 × 104 ml^−1^ were cultured on each sample for 24 h. After 1 day, each sample was transferred to a new 24-well plate to remove the cells attached to culture plate, not to the sample. The cultured cells were assayed with cell counting kit-8 (CCK-8, Dojindo molecular technologies, USA) solution, and the plate was incubated for 1 h with 10 vol% CCK-8 with media solution and measured at 450 nm with a microplate reader (EMax microplate reader, Bucher Biotec AG, Basel, Switzerland) for 1, 3, and 7 days after seeding. Four specimens from each group were investigated to derive the average values, and the control was the as-received substrate.

To evaluate cell spreading and shape, cells were fixed with 4% formaldehyde and phosphate-buffered saline (PBS) solution. Thereafter, the cells were permeabilized with cold acetone, washed three times with PBS solution and incubated with 4% Texas red phalloidin (Sigma-Aldrich, USA) in PBS solution for 30 min. After incubation and additional washing, cells were labeled with DAPI staining (Vector laboratories Inc., USA) and mounted for fluorescent imaging. Fluorescence images were obtained with confocal microscopy (Leica TCS-SP5-MP-SMD, Leica microsystems Wetzlar, Germany). The area of cell adhesion was measured with the image J software (image J, Sun Microsystems Inc., USA). Ten cells from each substrate were examined to evaluate the average area per one cell.

The as-received Ti with an adequate grain size for the experiment was used to study the etching effect on various orientations. At the outset, the microstructure and orientation of each grain were examined with the EBSD (OIM4000, EDAX Japan K.K., Japan) and OIM software (TSL OIM analysis 5.2, EDAX Inc., USA). Subsequently, etching was performed on the as-received surface for 20 min., and the surface roughness and topography of the substrate were assessed with AFM and optical profilometry. The same region on the substrate was evaluated to investigate the interrelation between orientation and roughness. The equipment, software, and etching method were the same as the ones mentioned above. Afterward, the cells were cultured for 3 days with the same process as mentioned before and were pictured by confocal microscopy. The area of cell adhesion was measured with the image J software. Average cell sizes in three grains (grains 1, 3, and 5 in Fig. 5) were evaluated.

### Statistical methods

All data were evaluated with the analysis of variance (ANOVA), and Student’s *t*-test was performed for valuations between groups. The significance level was set at *p* < 0.05.

## Additional Information

**How to cite this article:** Baek, S. M. *et al*. Superior Pre-Osteoblast Cell Response of Etched Ultrafine-Grained Titanium with a Controlled Crystallographic Orientation. *Sci. Rep.* **7**, 44213; doi: 10.1038/srep44213 (2017).

**Publisher's note:** Springer Nature remains neutral with regard to jurisdictional claims in published maps and institutional affiliations.

## Supplementary Material

Supplementary Information

## Figures and Tables

**Table 1:** Grain sizes and mechanical properties of the Ti samples.

	Grain size (μm)	UTS (MPa)	Uniform elongation (%)	Total elongation (%)
As-received	118 ± 49	540 ± 3	15 ± 1	56 ± 1
HPT 5 GPa 5 Turns	~0.1	1150 ± 29	5.5 ± 0.5	18 ± 1
HPT + Annealing 550 °C 2 h	3 ± 1	634 ± 5	16.5 ± 1.5	43 ± 3
Ti-6Al-4V^a^	—	860–1020	—	10–15

^a^References [5, 28].

**Table 2:** Surface roughness parameters and surface area differences of the Ti samples.

Materials	Optical profilometry			AFM
	R_a_ (nm)	R_q_ (nm)	R_max_ (μm)	Surface area difference (%)
As-received	27 ± 2	34 ± 3	0.4 ± 0.04	0.02 ± 0.01
eCG	146 ± 8	190 ± 10	2.5 ± 0.3	2.35 ± 0.6
eFG	91 ± 8	125 ± 11	2 ± 0.2	1.38 ± 0.4
eUFG	209 ± 15^***,†,‡^	279 ± 20^***,†,‡^	3.2 ± 0.1^***,‡,**^	3.37 ± 1^*^

R_a_ is the average roughness of the absolute value of the profile height; R_q_ is the root mean square roughness of surface; R_max_ is the height of the highest peak in the roughness profile. ^*^ if *p* < 0.05, and ^***^ if *p* < 0.001 compare with the as-received substrate; ^**^ if *p* < 0.01, and ^†^ if *p* < 0.001 with respect to the eCG substrates; and ^‡^*p* < 0.001 compared to the eFG substrates. There is no statistically significant difference in surface area difference between the eUFG, eFG, and eCG.
