# Supplementary material for: Superior Pre-Osteoblast Cell Response of Etched Ultrafine-Grained Titanium with a Controlled Crystallographic Orientation
Source: Sci Rep. 2017 Mar 7;7:44213. doi: 10.1038/srep44213 (PMC5339782; doi:10.1038/srep44213)
Supplement: Supplementary Information [file srep44213-s1.pdf]

Supplementary Information for

Superior Pre-Osteoblast Cell Response of Etched Ultrafine-Grained Titanium with a Controlled Crystallographic Orientation

SEUNG MI BAEK[1], MYEONG HWAN SHIN[1], JONGUN MOON[1], HO SANG JUNG[1,2], SEE AM LEE[1], WOON-BONG HWANG[3], JONG TAEK YEOM[2], SEI KWANG HAHN[1], HYOUNG SEOP KIM[1,4,*]

[1]*Department of Materials Science and Engineering, Pohang University of Science and Technology (POSTECH), Pohang, 37673, Republic of Korea*

[2]*Korea Institute of Materials Science, Changwon, 51508, Republic of Korea*

[3]*Department of Mechanical Engineering, Pohang University of Science and Technology (POSTECH), Pohang, 37673, Republic of Korea*

[4]*Center for High Entropy Alloys, Pohang University of Science and Technology (POSTECH), Pohang, 37673, Republic of Korea*

*Corresponding Author: Hyoung Seop Kim

E-mail: hskim@postech.ac.kr, Tel. +82 54 279 2150, Fax +82 54 279 2399

Supplementary method

Grain size measurement.

The grain size was measured with electron back-scatter diffraction (EBSD) and transmission electron microscopy (TEM). To investigate the grain sizes of the as-received and annealed specimens, EBSD (OIM4000, EDAX Japan K.K., Japan) was used with field emission scanning electron microscopy (FESEM: XL30S FEG, Philips electron optics B.V., Netherlands). Samples were prepared by mechanical polishing down to 0.25 μm using diamond pastes and electro-etched in an etchant of 6% perchloric acid, 35% butyl alcohol, and 59% methanol by volume. EBSD orientation image maps were acquired on the normal plane of the samples with a total of at least 2000 grains. The grain sizes were determined with orientation image microscopy (OIM) software (TSL OIM analysis 5.2, EDAX Inc., USA). The grain size of the HPT-processed specimen was examined by TEM (JOEL JEM-2200FS, JEOL, USA) on a jet-polished 3 mm disk thin foil.

Supplementary Figures

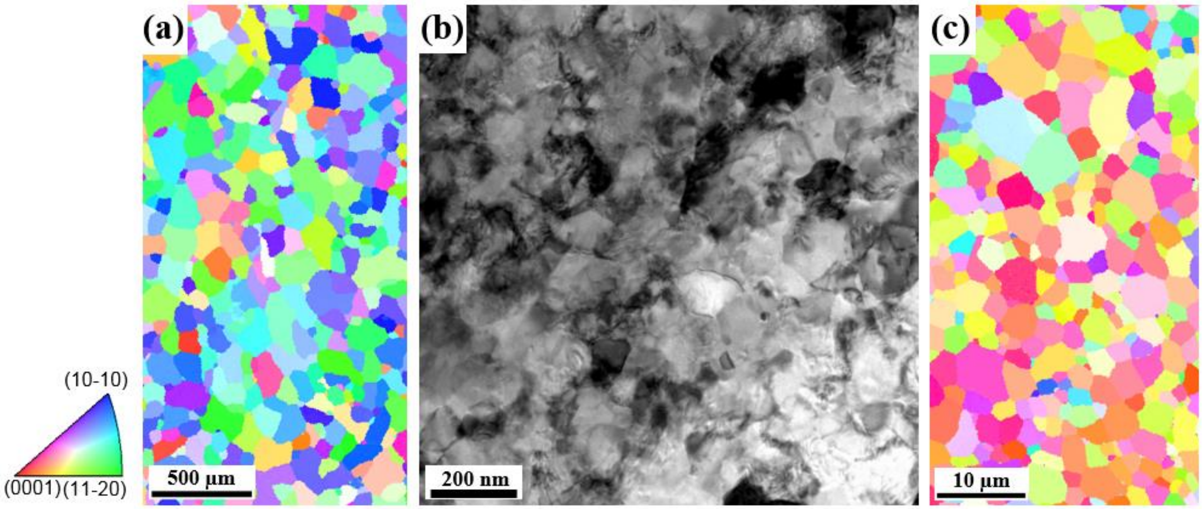

Figure S1. Microstructures of CP-Ti samples

EBSD micrographs of (a) the as-received and (c) HPT + annealed samples and (b) STEM micrograph of the HPT-processed sample
